# Statistical learning models to measure the impact of COVID-19 on financial fragility

**DOI:** 10.3389/frai.2024.1358812

**Published:** 2024-05-15

**Authors:** Arianna Agosto, Paolo Giudici, Alessandra Tanda

**Affiliations:** Department of Economics and Management, University of Pavia, Pavia, Lombardy, Italy

**Keywords:** COVID-19, households, financial vulnerability, financial fragility, statistical learning

## Abstract

This paper investigates the effects of the economic shock produced by the COVID-19 outbreak and diffusion on households'. Through a survey administered to Italian households, without loss of generality, we investigate changes in financial and economic decisions and the households' ability to cope with daily purchases, repay their debt obligations and face unexpected expenses. The paper also applies a statistical learning model through a synthetic indicator for the financial vulnerability of households, integrating the relevant information on the financial literacy and education of the surveyed individuals.

## 1 Introduction

Households can be exposed to external shocks that affect their ability to meet their daily expenses and influence their economic and financial decisions. According to the Bank of Italy's Annual Report 2021, the impact of COVID-19 on households was extremely diversified (Banca d'Italia, 2021). Despite the efforts of fiscal policy to support the income shock of households, a part of the latter was extremely affected in terms of income, which translated into a reduction in consumption and had an impact on economic and financial decisions. Economic factors have contributed to the reduction in consumption, especially among retired persons and the unemployed.

Following the COVID-19 outbreak, numerous studies have been conducted to investigate the impact of the pandemic on households (see Padhan and Prabheesh 2021; Rathnayaka et al. 2023, for a review). These studies have examined safety, health (Kansiime et al., 2021; Lecoutere et al., 2023) and the economic effects on family businesses (Baker et al., 2020) or start-up creation (Albert et al., 2023) in both advanced (Jung et al., 2023) and emerging economies (Mallawaarachchi and Rahut, 2023).

Among the studies, only a few specifically address the impact of the pandemic on economic and financial decisions by households. Yue et al. (2020) study the effects of COVID-19 on investment decisions in China and find that households generally decreased the invested amount as an effect of a more risk-averse attitude. Jia et al. (2022) also focus on Chinese households. The study shows that the investment behavior of households underwent significant changes before, during, and after the pandemic. These changes were caused by the altered expectations resulting from the spread of COVID-19. Yazdanparast and Alhenawi (2022) evaluate the impact of COVID-19 on financial decision-making and find that both personality traits and environmental factors play a role in shaping financial decisions.

This paper presents a statistical learning model employing a synthetic indicator of households' financial vulnerability by integrating information from a 49-question questionnaire that investigates how spending and financial habits have been affected by the COVID-19 outbreak. The analysis also reveals the primary determinants of financial vulnerability in households. The study examines whether education level, financial literacy, and confidence with financial instruments can reduce financial vulnerability. The results indicate that the previous use of financial instruments is the most significant variable. The paper is structured as follows: Section 2 describes the Research design; Section 3 presents the model employed; Section 4 illustrates the main evidence and results; Section 5 concludes.

## 2 Research design

The survey consisted of 49 questions (See Box 1). To improve the response rate among Italian households, the survey was conducted in Italian.

Box 1 **Survey (English translation): main questions.**

- D1-Gender: *M; F; other/prefer not to respond*
- D2-Age: *in years*
- D3-Family members: *1; 2; 3; 4; or more*
- D4-Children: *0; 1; 2; 3; 4; or more*
- D5-Co-habitants/Family structure status: *I live alone; young couple* *with no kids; elderly couple with no kids; I live with young children* *(*<*18 years); I live with* *older children (*>*18* *years); I live with my parents; I live with my child's family; I live with other people*
- D6-Marital status of the respondent: *single; cohabitant; married; widow; separated;* *divorced*
- D7-Postal code
- D8-Educational level of the respondent: *Post-graduate degree; Bachelor degree or equivalent;* *Upper secondary school; Junior secondary school; Elementary; None*
- D9-Is the house you live in currently living in ...? *Property; Rented; Owned by third parties* *(non-cohabitants) in free use*
- D9.1-Do you have a loan for the purchase of the residence? *Yes; No*
- D9.2-How much is the installment your family pays in a month for the mortgage or mortgages? *Up to 200 euros per month; 201–400; 401–600; 601–600; 801; 1,000; 1,001–2,000; more than* *2,000 euros per month*
- D10-currently in the family you are paying the installments of a loan (consumer credit, personal loan, salary-backed loan) [for all questions, answers: *yes; no*]
  - D10.1-for the purchase of furnishings; furniture or household appliances (TV, washing machines, computers, ...)?
  - D10.2-for the purchase of cars, motorcycles, other vehicles?
  - D10.3-If you answered “yes” to one of the two previous questions, on average how much is the monthly payment that your family pays in a month for non-mortgage loans (consumer credit, personal loan, salary-backed loan)? *Up to 200 euros per* *month; 201–400; 401–600; 601–600; 801; 1,000; 1,001-2,000; more than 2,000* *Euros per month*
- D11-Now let's refer to your situation before and after Covid. For your family, the monthly payment related to the previous application represented an expense/burden that you incurred with ... [March 2019–February 2020] & [March 2020–June 2021]: *much ease; easily; some difficulty;* *difficulty; much difficulty*
- D12-Have you requested a loan from banks or financial companies starting from March 2020 (start of lockdown measures)? *Yes; no*
  - D12.1-Financing has been granted? *Yes; no; partly*
  - D12.2-Which entity did you contact to request the loan? *bank; consumer loan* *or other specialized lending institution; crowdfunding or peer-to-peer lending* *platform; other*
  - D12.3-Through what channel? *branch; online; other*
- D13-Since March 2020, someone in the family has benefited from income support benefits—e.g., Unemployment benefits, mobility, family unit allowances, etc. or financial support also in the form of services? *Yes, by state / state institutions; Yes, by private or religious* *institutions (foundations, private entities, companies, etc.); No, we didn't benefited from* *it*
- D14-Now let's refer to your situation before and after Covid. At that time, the monthly income allowed you to reach the end of the month with ...? [March 2019–February 2020] & [March 2020–June 2021] *A lot of ease, manages to save enough; Ease, it also manages to save* *something; Some difficulties, we just manage to make ends meet; Difficulty, we had to dent* *savings; Much difficulty, we had to ask for help*
- D15-Now let's refer to your situation before and after Covid. In that period, would you have been able to incur an unexpected expense of 500 Euros? [March 2019–February 2020] & [March 2020–June 2021] *Yes, very easily; Yes, with ease; Yes, but with difficulty; Yes, but with great* *difficulty; No*
- D16-With reference to the period that started in March 2020 [for all questions, answers: *yes, as* *in the past; yes, for the first time; no*]
  - did you have difficulty in supporting daily expenses (food, supermarket shopping)?
  - have you had difficulty paying utility bills (electricity, gas, telephone, etc.)?
  - have you had difficulty paying the rent or the home loan?
  - have you had difficulty repaying the loan installments (other than the mortgage if present)?
  - did you have to forgo a specialist medical examination or therapeutic treatment for economic reasons?
  - have you had to renounce for economic reasons to take out non-compulsory insurance policies (e.g., accidents, illness, and life/death cases)?
- D17-With reference to the period that started in March 2020, have you postponed investments or expenses already planned for economic reasons? *yes, car or motorbike purchase; yes, main* *house purchase; yes, other real estate purchase; Yes, different or non-essential goods or* *services purchase (TV, computer, holidays, sports, personal training courses ...);* *No*
- D18-Since March 2020, have you lost your job (or any of your cohabiting family members lost it)? *Yes; no; prefers not to answer*
  - D18.1-If you answered yes to the previous question, the loss of income due to the dismissal has had an impact: *very high; high; medium; relatively small; small*
- D19-Since March 2020, has an illness involved you or your family members leading to an unexpected reduction in income or an unexpected increase in family expenses? *yes;* *no*
- D20-Since March 2020, has a death involved you or your family members resulting in an unexpected reduction in income or an unexpected increase in household expenses? *yes;* *no*
- D21-Current profession of the respondent: *student; Houseperson; Retired; Unemployed;* *Manager, officer, middle manager; Office worker, teacher, military; Worker, clerk, apprentice;* *Entrepreneur, freelancer; Merchant, craftsman, farmer; Atypical work (occasional collaboration*, *project work, etc.)*
  - D21.1-If unemployed: what activities did you carry out before being unemployed? *Student; Houseperson; Retired; Manager, officer, middle manager; Office worker*, *teacher, military; Worker, clerk, apprentice; Entrepreneur, freelancer; Merchant*, *craftsman, farmer; Atypical work (occasional collaboration, project work, etc.)*
- D22-Approximately what range does your household's total monthly net income fall into? *up to 600 Euros; 601–1,200 Euros; 1,201–2,000 Euros; 2,001–3,000 Euros;* *3,001–4,000 Euros; 4,001–5,000 Euros; more than 5,000 Euros; don't know; prefers not to* *answer*
- D23-I use financial instruments and products [for all questions, answers: *yes; no*]
  - D23.1-Do you own a current account?
  - D23.2-Do you own at least one credit card?
  - D23.3-Do you own any government bonds or other bonds?
  - D23.4-Do you own shares or funds/SICAVs?
- D24-Imagine that you have a debt of 1,000 Euros on your credit card and that the interest rate applied is 20% (compound annual rate). If you pay nothing, at this interest rate, how many years would it take to double the amount owed? *2 years; < 5 years; 5–10 years; don't* *know*
- D25-You have a debt of 3,000 Euros for the use of your credit card. Pay an installment of 30 Euros each month. At an annual rate of 12% (or 1% monthly), how many years would it take to eliminate credit card debt if you don't make any further purchases? *less* *than 5 years; 5–10 years; 10–15 years; Never, will you continue to be in debt; don't* *know*
- D26-You buy a product that costs 1,000 euros. To pay, you have the following two options: (a) pay 12 monthly installments, each of 100 Euros; (b) borrow at an annual interest rate of 20% and pay back 1,200 euros in a year. Which option is more advantageous? *option a; option b;* *indifferent; don't know*
- D27-On a scale of 1 to 5, where 1 means very low and 5 means very high, how would you rate your knowledge of financial topics? *1(Very low); 2; 3; 4; 5(Very high)*
- D28-On a scale of 1 to 5, how well do you feel you are able to make good decisions? *1(not good* *at all); 2; 3; 4; 5(very good)*

The questionnaire comprises questions to understand the demographic profile of respondents (e.g., age, gender, marital status, etc.), the educational level and the current wealth and debt situation. For example, respondents were asked if they are currently repaying loans for their house or car.

Respondents are then asked to assess their situation before and after the outbreak of COVID-19. The aim is to assess any potential increase in financial difficulties, not only in relation to loan repayments but also in everyday expenses. Additionally, questions are posed to test respondents' financial competence and experience in financial investments, as indicated by the number of different financial and investment products invested. The final two questions explore respondents' perceived knowledge of financial topics and their ability to make sound decisions.

## 3 Modeling framework

This paper provides a synthetic indicator for the financial vulnerability of households, by integrating the information retrieved from the questionnaire presented in Section 2.

The survey responses are transformed into categorical variables. capability of explaining the possible occurrence of financial distress. The target variable, representing a respondent's spending capacity, is modeled as a binary variable that is equal to 1 if the respondent has experienced a deterioration in spending capacity compared to the pre-pandemic period (see Section 4 for details on the definition of the target and predictor variables obtained from the questionnaire).

Let us denote with

- θ_*i*_ the probability of financial distress occurrence for the household *i* conditional on the available covariates;
- *X* the *K*-dimensional set of covariates *X* retrieved from the questionnaire. Each household belongs to a class *j* of the categorical *X*_*k*_ variable;
- *d*_*j*_ and *n*_*j*_ the number of distressed and non-distressed households in class *j* of the covariate, respectively.

Following Agosto et al. (2023a,b), the expected probability θ_*i*_ of financial distress occurrence for household *i* is obtained as reported in Equation (1).


(1) $$\begin{array}{l} E\left(\theta_{i}|X,Y\right)=\overset{K}{\underset{k=1}{\sum }}E\left(\theta_{j}|g_{k},Y\right)p\left(g_{k}|Y\right), \end{array}$$

with $E\left(\theta_{j}|g_{k},Y\right)=\frac{\alpha +d_{j}}{\alpha +\beta +n_{j}}$,

where the α and β parameters are set equal to the frequency of distressed and non-distressed households in the sample respectively, and *p*(*g*_*k*_|*Y*) is the marginal contribution of the *k*-th variable to the likelihood of financial distress occurrence, calculated using the Bayesian methodology proposed by Agosto et al. (2023a).

Such contribution acts as *k*-th covariate weight in determining the expected probability of financial distress occurrence defined in Equation 1. Its magnitude depends on the capability of the partition *g*_*k*_ generated by the *k*-th categorical variable in predicting the binary target variable which expresses the household's financial vulnerability. Note that the approach relies on the assumption that the probability of financial distress is constant within the same *j* level of the *k*-th covariate.

This methodology – relying on the theoretical framework of Giudici et al. (2003) and proposed in the credit risk context by Cerchiello and Giudici (2014)—allows leveraging the information coming from categorical indicators by finding their optimal combination based on predictive accuracy *blue* measured on a training dataset. Indeed, the weights assigned to the covariates are used to combine them into a “superscore,” to predict households' financial vulnerability.

Potentially, in contexts where more data are available, the data-driven merge of different information sources could also be performed using artificial intelligence techniques, as done in Agosto et al. (2023a), where XG-BOOST was considered as a benchmark model. It is important to remark that our statistical learning methodology is explainable by design and offers a system of weights that can be used in further analyses, while boosting methodologies can only be explainable ex-post and using computationally expensive artificial intelligence methods, such as Shapley values.

## 4 Empirical study

### 4.1 Data

The survey was conducted between June 2021 and June 2022, and 58 valid responses were collected during this period^1^. The gender distribution of the respondents is relatively equal, with 52% identifying as male, 47% as female, and one respondent not providing their gender.

The majority of respondents are under 30 years old. Answers are based on family decisions, so age is not considered a relevant variable. The surveyed households mostly consist of families with four members. Around 67% of households consist of three or more members. Approximately 21% of households are comprised of three members, 5% of households have two members, and the remaining 7% are single-member households. In terms of assets, most households own their homes, with around one-third having an ongoing mortgage. The monthly installment amount varies, but the majority pay less than 600 euros per month. A few families also have consumer or personal loans.

### 4.2 Results of the survey

**Figure 1:**
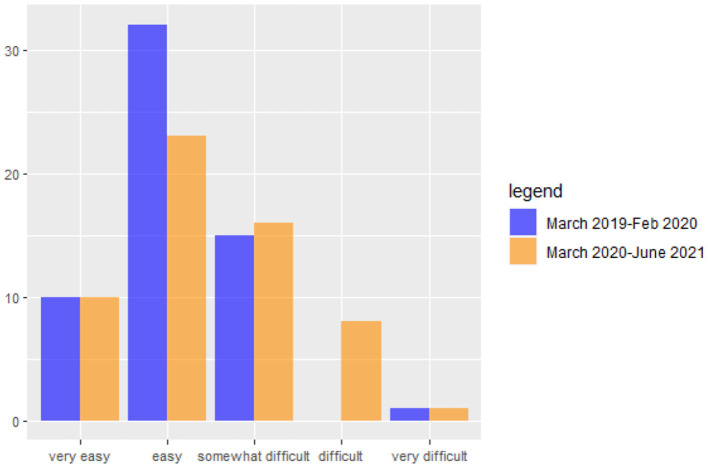
Number of families experiencing difficulties in paying the monthly installments before and after Covid outbreak.

The COVID-19 outbreak has affected households' ability to manage expenses and make mortgage and loan payments. Figure 1 illustrates that during the COVID-19 period, the number of families experiencing difficulty paying their monthly installments has increased. Specifically, there has been an increase in the number of families finding it “somewhat difficult” and an increase from 0 to 8 families finding it “difficult.” The families at the extremes of the distribution, whether very easy or very difficult, did not experience any change (see Figure 1).

**Figure 2:**
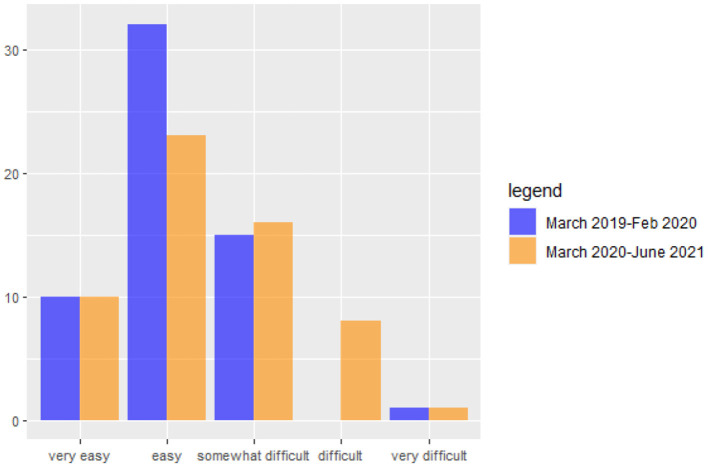
Ability of families to make ends meet before and after Covid outbreak.

When assessing whether families can make ends meet, we observe an increase in the number of families experiencing financial difficulties and a corresponding decrease in those able to save (Figure 2). Similarly, the number of families capable of comfortably handling an unexpected expense of 500 euros decreased after the COVID outbreak. Some families were forced to forgo purchases related to daily necessities such as food (6), utilities (10), health or medical expenses (7), or insurance (6). Approximately 30% have postponed non-essential expenses such as TV, entertainment, or car/motorbike purchases.

### 4.3 Financial vulnerability factors

A statistical analysis was conducted to elucidate the factors influencing financial vulnerability during the pandemic. The study specifically investigates the potential roles of education level and financial literacy in mitigating the economic impact of pandemics on households.

The questionnaire answers are categorical variables from a statistical perspective. Some indicators of financial distress include the ease of making ends meet, while predictive variables can include self-evaluation of financial knowledge. The definition, type and source of the variables used in this empirical study are reported in Table 1.

**Table 1:** Definition of the main variables used in the study.

**Variable**	**Type**	**Source**
Capability of affording an unexpected expense	Categorical	Survey (D15)
Capability of making ends meet	Categorical	Survey (D14)
Education level	Categorical	Survey (D8)
Financial literacy	Categorical	Survey (D24 to D28)
Confidence with financial instruments	Categorical	Survey (D23)

We, thus, apply the methodology presented in Section 3 to combine the available information on household features and behaviors in a “financial vulnerability” index, which should reflect the household's exposure to the potential financial impact of pandemics. The potential weakness factors that we consider are related to low educational level, low financial literacy and low confidence with financial instruments. Specifically, starting from the variables described in Table 1, we build the following binary indicators:

- binarized education level: a binary variable which is equal to 1 if the respondent has an education level lower than degree, 0 otherwise;
- binarized financial literacy: a binary variable which is equal to 1 if the respondent has given less < 2 correct answers, 0 otherwise;
- binarized confidence with financial instruments: a binary variable which is equal to 1 if the respondent holds no more than one instrument among credit cards/stocks/government or other bonds, 0 if the held instruments are more than one.

Then, using the variable “Capability of affording an unexpected expense” (see Table 1), we define our target variable as a binary one, which is set equal to 1 if the respondent had a worsening in the capability of affording unexpected expenses with respect to the pre-pandemic period. Based on the collected data, nearly 34% of respondents had a worsening in the spending capacity due to the pandemic.

For each of the three predictors, using the methodology presented in Section 3, we calculate a weight, based on the variable's accuracy in predicting the target, i.e., the possible deterioration in the respondent's spending capacity. The estimated weights are reported in Table 2.

**Table 2:** Weights of the financial vulnerability index components.

**Variable**	**Estimated weight**
Education level	22%
Financial literacy	23%
Confidence with financial instruments	55%

The weights are then used to calculate the financial vulnerability index. For example, if an individual has a score of 0 in “educational level,” 1 in “financial literacy” and 1 in “confidence with financial instruments,” the financial vulnerability index will be calculated as: 0*0.22 + 1*0.23 + 1*0.55 = 0.78. Preliminary analyses—not shown here for brevity—revealed a positive relationship between each of the three dummy variables and the target variable. Thus, the individual will be considered as more financially vulnerable than, for instance, an individual which was assigned an index of 0.7. As explained in Section 3, the index can be interpreted as a superscore combining different aspects related to financial fragility.

Similar results (with weights equal to 21%, 21%, and 58%) were obtained by using, as the target variable, deterioration in the ease of reaching the end of the month, a binary variable set equal to 1 if there was a worsening in the “Capability of making ends meet” variable (see Table 1). Indeed, nearly 31% of respondents declared a worsening in the capability of making ends meet in the pandemic and post-pandemic period.

## 5 Conclusions

This paper assesses the impact of the COVID-19 outbreak on the economic and financial decisions of Italian households. Using a statistical learning model we show that COVID-19 affected the ability of households to manage expenses and repay mortgage and loan installments, particularly those who were already in a financially fragile state.

A statistical analysis is conducted to identify the drivers of financial vulnerability in the sample. The evidence suggests that financial vulnerability is influenced by confidence with financial instruments. On the other hand, education level and financial literacy have a lower effect. Similar results were found when using the inability to make ends meet as the target variable.

## Data availability statement

The original contributions presented in the study are included in the article/supplementary material, further inquiries can be directed to the corresponding author/s.

## Ethics statement

The study involving human participants was approved by the Independent Ethics Advisory Board of the PERISCOPE Project. The research was conducted in accordance with the local legislation and institutional requirements. The participants provided their explicit informed consent to participate in this study by filling in the survey.

## Author contributions

AA: Conceptualization, Data curation, Formal analysis, Investigation, Methodology, Validation, Writing – original draft, Writing – review & editing. PG: Conceptualization, Funding acquisition, Methodology, Project administration, Supervision, Validation, Writing – original draft, Writing – review & editing. AT: Conceptualization, Data curation, Investigation, Supervision, Visualization, Writing – original draft, Writing – review & editing.

## Footnotes

^1^To preserve the privacy of respondents, data are anonymously collected and are available upon request from the author only at an aggregated level.
